# The Tissue Changes in Diphtheria

**Published:** 1908-04-04

**Authors:** 


### Pathology.

### THE TISSUE CHANGES IN DIPHTHERIA.

It must always be helpful, in the treatment of a patient, to know exactly what is going on in his or her tissues; hence every exact research into pathology is of value to medical men. It is a pity, perhaps, that pathological investigation of recent years has been concentrated so much upon bacterial work, and that researches into microscopical and chemical pathology have been in comparative abeyance; this, however, is beginning to be corrected, and Dr. Dudgeon has published in the St. Thomas' Hospital Reports some very instructive work upon the various changes which occur in the tissues in acute diphtheritic toxæmia.

The text-books give but very brief accounts of the effects of diphtherial toxins on the various organs in the body, and the descriptions are usually too vague to allow of their being turned to direct account in connection with treatment. Dr. Dudgeon's work throws a much more definite light upon the subject, and we are able to deduce from it more than one point of practical importance, as will be seen in the summary at the end of this review.

We need not enter fully into the methods of investigation employed, beyond saying that the work was carried out not only by the injection of diphtheria toxins into animals, but also by histological examinations of the organs of patients dying directly from diphtheria; and, further, that instead of osmic acid being used as the chief stain for the detection of fat particles and globules, two other stains were used as well, namely Sudan III. and Scharlach R. We have elsewhere referred to the great advantages either of these stains has over osmic acid in the detection of very small globules of fat.

Text-books as a rule make little attempt to distinguish between the changes which occur during the acute diphtheria, and those which may be found should death occur later as the result of a complication or sequela—bronchopneumonia, for example, or pericarditis, or peripheral neuritis, and so forth. We are at present dealing solely with the changes which occur in the tissues during the uncomplicated and acute diphtheria. We will first enumerate the tissues in which no fatty changes are found. These are: The skeletal muscles (not including the diaphragm), tongue, œsophagus, stomach, duodenum, jejunum, ileum and colon, the bladder, the pancreas, the thyroid gland, the thymus gland, the lymphatic glands, the blood, the pleural fluid, the brain, and the phrenic and the vagus nerves.

A little explanation may be necessary in regard to the brain, and the phrenic and vagus nerves. These may show degenerative changes later on, when the acute diphtheria is past. During the ten days or fortnight that the diphtheria may be called acute, however, the changes in the brain and these two nerves are scarcely recognisable under the microscope, whereas, as we shall see, the changes in the heart and in the diaphragm may be very great in a much shorter time than ten days. It would seem, therefore, that the diphtheria toxin, whether it also acts through the vagus nerve or not, has a direct and deleterious action on the protoplasm of the cardiac muscle.

The tissues which show definite changes, both in the human cases and in the animals experimented upon, are the following: The heart, the diaphragm, the kidneys, the liver, the suprarenal glands, the cells of cartilages, and the spleen. The change in each case is a fatty one, and without discussing the vexed question as to whether it is a fatty degeneration or not, we will be content to call it "fatty change," although we believe the evidence is strongly in favour of it really being a conversion of the cell protoplasm into fat, in other words a true fatty degeneration. We can dismiss as relatively unimportant the fatty changes in the spleen and in the cartilage cells, but we must discuss more fully those in the heart, the diaphragm, the suprarenal glands, the liver and the kidneys.

There is little doubt that the gravest group of symptoms which occur in diphtheria are those which are described as "*cardiac paralysis.*" This has led to the investigation of the condition of the heart-muscle by many observers, but seldom has there been a full examination of the other organs microscopically at the same time. Moreover, the heart itself has not always been investigated histologically, and to the naked eye it has often appeared normal. The result has been that the condition which has led to death has come to be termed cardiac *paralysis*, an expression which is apt to convey the idea that the fatal trouble is not in the heart-muscle itself, but in the nerves controlling the heart. This is a pity, because the evidence is now pointing towards the fatty changes in the heart-muscle ante-dating any affection of the nerves; it would be better, therefore, to speak of the condition as an *acute cardiac failure from fatty change* than as a paralysis; in addition to which it must now be recognised that the changes which occur simultaneously in the liver and in the suprarenal glands, as well as elsewhere, have almost certainly a considerable effect upon the patient also and upon the vigour of the heart's action.

The fatty changes in the heart-muscle consist chiefly in the appearance throughout each muscle fibre of minute granules of fat, almost too small to be called droplets, but very distinctly seen under the high power when the section has been stained by Sudan III., or Scharlach R. Although these diphtheritic hearts show such very m[illegible] fatty change histologically, yet macroscopically there is little to detect beyond the fact that they are usually soft and flabby. There is not any "tabby-cat" striation such as is seen in fatal cases of pernicious anæmia where the fat globules in the fibres are comparatively large and unevenly distributed. It is often impossible to give an accurate opinion of the changes which may be present without microscopical evidence.

It has been recorded that the fatty changes in the heart may be found as early as the third day of the diphtheria. The earliest date at which Dr. Dudgeon himself has seen it is the fourth day of the disease in man, and in this particular case the fatty change in the myocardium was already quite extensive by the fourth day. In his animal experiments, however, it occurred within sixteen hours, in other words in so short a time that it is almost impossible not to believe that the change is due to the action of the diphtheria toxin directly upon the heart-muscle. When the fibres were found to contain scattered fat particles the remainder of the protoplasm might be comparatively healthy-looking, or it might be generally degenerate, with loss of transverse striation, swollen, distorted or non-staining nucleus, a tendency to fragmentation and segmentation, and even vacuolation.

Control examinations were made upon the heart-muscle of cases dying of other diseases than diphtheria, in order to be sure that the fatty changes were not due to post-mortem conditions. One of the control cases was that of a woman aged 40, who died from acute oxalic acid poisoning; this heart showed an extensive diffuse fatty change throughout the muscle fibres, precisely similar to that of acute diphtheritic toxæmia; none of the others showed anything like it, so that it was clear that the fatty changes were no post-mortem artefacts, though other lesions besides diphtheria might produce them.

Weakness or paralysis of the *diaphragm* is very seldom seen during the acute stages of diphtheria, so that the muscle continues to do its work normally, although it is diseased. Notwithstanding the fact that contractions of the diaphragm continue, Dr. Dudgeon has found that it shows the acute degenerative changes even earlier than the heart-muscle does, and in some instances diffuse fatty change may occur while the myocardium is apparently normal. This fact in itself serves to prove the most important point in diphtherial toxæmia, namely that the toxins act directly upon the tissues, such as the heart and diaphragm, and not indirectly through the nerves. A guinea-pig injected with diphtheritic toxin showed fatty change in the muscle fibres of the diaphragm four hours after inoculation, before any other recognisable change had occurred beyond congestion of the suprarenal glands. The common type of fatty change consists of very fine droplets distributed in groups throughout the protoplasm of the fibre. Coarse fat droplets are completely absent. It is remarkable that the diaphragm shows such early and such marked fatty change when the striped muscles of the limbs show none at all.

The *liver* seems to be especially prone to suffer from the effects of the diphtheritic toxin. Dr. Dudgeon found the hepatic tissue in one case to consist of little more than fat droplets, while in most instances the fatty change was well marked. Occasionally the fatty change is so great in diphtheria that the liver is pale yellow throughout, and has so low a specific gravity that it floats. The fat droplets are particularly abundant in the areas surrounding the hepatic veins; and in the peripheral portions of the cells.

The fatty change in the *kidney* present to some extent in almost all cases, is best observed in the epithelium of the convoluted tubules, though none of the renal epithelium was exempt. Pathological changes in the blood-vessels, and areas of interstitial inflammation, do not accompany the fatty changes in the parenchyma even in those instances where albuminuria has been noted during life.

The most characteristic lesion produced by the diphtheritic toxins in guinea-pigs is congestion of the *suprarenal glands*; the change can be detected as early as four hours after an injection. It may become extreme, so that the gland is a mere capsule of blood. Human beings who have died of diphtheria do not show this acute congestion to anything like the same extent, but they do show extensive fatty changes. This is a very important point.

The normal suprarenal contains some fat, confined to the cortex, and chiefly in its outer parts. The medulla contains no fat in health. Guinea-pigs killed by the diphtheritic toxin present a great excess of fat, not only in the cortical cells of their suprarenals, but also throughout the medulla. Children who have died of diphtheria exhibit a similar fatty change in the medulla of their suprarenals as well as in the cells of the cortex. Dr. Dudgeon very pertinently lays stress on this; for it is the function of the medullary portion of these glands to produce adrenalin, and supply it to the other tissues of the body, particularly to the heart and blood-vessels, to increase their tone and contractile force. Without an adequate supply of this internal secretion of the suprarenal glands life is not possible. The diphtheritic toxin appears to be particularly prone to produce severe pathological changes in them, and the lack of proper suprarenal activity is very likely as serious a factor in the acute heart failure as is the fatty change in the myocardium itself.

The effect of *anti-diphtheritic serum* given as early as possible in the illness is an immense diminution in the degree of fatty change that the above organs undergo. This is, no doubt, because the anti-toxin combines with the toxin, and neutralises it before it exerts its baneful influence on the protoplasm of the heart, liver, diaphragm, kidneys, or suprarenal glands. To wait before giving the anti-toxin is to shut the stable door after the horse has run away. The fatty changes are in full swing by the fourth day. One experiment will indicate how beneficial the anti-toxin is if given early. Dr. Dudgeon found that one guinea-pig which received 160 times the minimum lethal dose of virulent toxin, and also 2,000 units of anti-toxin at the same time, recovered completely after an illness of a few days. Killed twenty-five days later, the only organs in the body that showed fatty change were the suprarenal glands, and even these were not extensively affected. Without the anti-toxin a minute portion of the dose of toxin given would have caused the fatty changes in all the organs described above.

The following conclusions are suggested by the paper:—

Diphtherial toxin acts directly on the protoplasm, not only of the heart, but also of the liver, kidneys, diaphragm, and suprarenal glands. Its action is very rapid. The changes in all these organs are summarised by the word "fatty," and they may be well marked within four days from the beginning of the diphtheria. The fat particles are very small, and the organs may be passed as normal if examined only by the naked eye. Sudan III. is a better stain than osmic acid. Cardiac paralysis is a bad term, because it implies a nerve lesion, whereas the most obvious cardiac affection is fatty change in the myocardium. The deficient power of the heart is made much worse by the lack of proper activity in the suprarenal glands, which become fatty at the same time. The general metabolism of the body must also be affected by the fatty changes in the cells of the liver and of the kidney.

The changes occur in every case experimentally inoculated; they therefore in all probability occur in every human case to a greater or a less degree—recovery is no proof that there have been no fatty changes. Convalescence must therefore include the clearing-up of fatty changes in several organs.

The giving of anti-toxin at the very earliest possible moment is *the* method of prophylaxis against the fatty changes in the organ; this is an additional argument in favour of giving it in every case, and not only in those which at the time seem to be severe. One should not wait till the condition is severe.

